# Clinical and Echocardiographic Factors Associated With AI‐Estimated Atrial Fibrillation Likelihood During Sinus Rhythm in Patients With Documented Paroxysmal Atrial Fibrillation

**DOI:** 10.1002/joa3.70439

**Published:** 2026-08-03

**Authors:** Hiroki Sato, Akiko Baba, Yuki Kubota, Nozomi Kodama, Kei Hirota, Miho Miyoshi, Hidekazu Kondo, Akira Fukui, Tomoko Fukuda, Tetsuji Shinohara, Yasushi Teshima, Naohiko Takahashi

**Affiliations:** ^1^ Department of Cardiology and Clinical Examination, Faculty of Medicine Oita University Yufu Japan; ^2^ Disaster Management Center Oita University Hospital Yufu Japan

**Keywords:** artificial intelligence electrocardiography, hypertension, left ventricular mass index, paroxysmal atrial fibrillation, tricuspid regurgitation

## Abstract

**Background:**

Artificial intelligence‐enabled electrocardiography (AI‐ECG) has emerged as a promising tool for identifying patients with atrial fibrillation (AF) using sinus‐rhythm ECGs. However, some patients with paroxysmal AF (PAF) may be assigned by AI‐ECG to lower AF likelihood categories. Clinical and echocardiographic characteristics associated with AF likelihood assignment by AI‐ECG remain unclear.

**Methods:**

This single‐center prospective study enrolled adults with documented PAF admitted for catheter ablation who were in sinus rhythm on the admission ECG. The four AF likelihood categories output by AI‐ECG were dichotomized into higher and lower groups for analysis. Logistic regression was used to assess factors associated with assignment by AI‐ECG to the higher AF likelihood group.

**Results:**

Among 104 patients, 36 were categorized into the lower and 68 into the higher AF likelihood group. Hypertension was associated with lower odds of higher‐group assignment (odds ratio [OR], 0.25; 95% confidence interval [CI], 0.10–0.66). In the echocardiographic model, higher left ventricular mass index (LVMI) was associated with lower (OR, 0.72 per 10 g/m^2^ increase; 95% CI, 0.57–0.90) and mild or greater tricuspid regurgitation (TR) with higher (OR, 2.99; 95% CI, 1.14–7.87) odds of higher‐group assignment.

**Conclusions:**

A lower AF likelihood assignment by AI‐ECG was not uncommon among patients with documented PAF. Hypertension and higher LVMI were inversely associated with assignment to the higher AF likelihood group, whereas mild or greater TR showed a possible positive association. These findings suggest that AI‐ECG‐based AF likelihood assignment varies among patients with documented PAF during sinus rhythm.

## Graphical abstract

Among patients with documented paroxysmal atrial fibrillation assessed during sinus rhythm, lower AF likelihood assignment by AI‐ECG was not uncommon. Hypertension and higher left ventricular mass index were inversely associated with assignment to the higher AF likelihood group, whereas mild or greater tricuspid regurgitation showed a possible positive association, suggesting heterogeneous AI‐ECG output within this population.

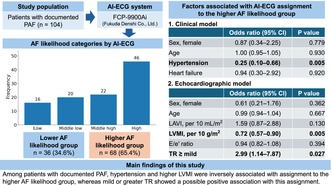

## 1 Introduction

Atrial fibrillation (AF) is the most common sustained cardiac arrhythmia and is associated with stroke, systemic thromboembolism, heart failure, and mortality [1]. Early recognition of AF is therefore clinically important. However, because AF can be paroxysmal and asymptomatic, a single 12‐lead electrocardiogram (ECG) recorded during sinus rhythm may fail to identify affected individuals [1, 2]. Although prolonged ECG monitoring can improve AF detection, its routine use is often limited by cost, logistical burden, and the need for specialized interpretation [2].

Artificial intelligence‐enabled electrocardiography (AI‐ECG) has emerged as a promising approach for identifying patients with paroxysmal AF (PAF) using standard 12‐lead ECGs recorded during sinus rhythm [3, 4, 5]. In the landmark study by Attia et al., an AI model applied to sinus‐rhythm ECGs achieved an area under the curve (AUC) of 0.87 for identifying patients with AF or atrial flutter [3]. Subsequent studies have shown reasonable diagnostic performance of AI‐ECG for identifying patients with underlying PAF using sinus‐rhythm ECGs [4, 5, 6]. If AI‐ECG is to be used for AF screening in clinical practice, it is also important to understand why some patients with PAF may nevertheless be assigned a lower AF likelihood when the ECG is recorded during sinus rhythm. Patients with documented PAF represent a clinically confirmed population that AI‐ECG‐based AF screening is intended to identify. Studying this population may help clarify limitations and interpretive considerations of AI‐ECG‐based AF screening. We therefore examined clinical and echocardiographic features associated with AI‐ECG‐based AF likelihood assignment in patients with documented PAF who were in sinus rhythm at the time of ECG recording.

## 2 Methods

### 2.1 Study Design and Population

This was a single‐center prospective observational study conducted at Oita University Hospital, Yufu, Japan. We prospectively enrolled consecutive adult patients with documented PAF who were admitted for catheter ablation between August 1, 2025, and March 31, 2026. Patients were eligible if they were in sinus rhythm on the admission 12‐lead ECG. Patients were excluded if AF likelihood assignment by AI‐ECG was unavailable.

### 2.2 AI‐ECG‐Based AF Likelihood Assignment

All patients underwent 12‐lead ECG on the day of admission for catheter ablation using the FCP‐9900Ai system (Fukuda Denshi Co. Ltd., Tokyo, Japan). The FCP‐9900Ai system is an AI‐enabled electrocardiograph designed to estimate the likelihood of PAF from 10‐s, 12‐lead ECG waveform data recorded during sinus rhythm. The AI model implemented in this system was originally developed using ECGs retrospectively collected from seven hospitals in Japan, including patients aged > 40 years with documented AF and those without documented AF. In the original development and validation study, 12‐lead sinus‐rhythm ECG waveforms were converted into binary images and analyzed using a lightweight convolutional neural network. The final datasets comprised 2330 ECGs for training and validation, 234 ECGs for internal testing, and 440 ECGs for external testing using data from two additional Japanese hospitals. The model showed AUCs of 0.82 and 0.80 in the internal and external testing datasets, respectively [7]. Based on the model output, the FCP‐9900Ai system assigns each ECG to one of four ordered AF likelihood categories: high (H), middle‐high (MH), middle‐low (ML), and low (L). The numerical thresholds defining these four categories are not publicly disclosed and were not available to the authors.

### 2.3 Clinical and Diagnostic Assessment

Demographic and clinical characteristics, including age, sex, medical history, comorbidities, and major medication classes, were obtained from the medical records. Laboratory data were obtained from blood samples collected at admission. Electrocardiographic variables were obtained from the 12‐lead ECG recorded at admission. Transthoracic echocardiographic data were obtained from outpatient examinations performed within 6 months before admission during a clinically stable period.

### 2.4 Procedural and Follow‐Up Variables

The presence of left atrial low‐voltage area was assessed during the ablation procedure and defined as an area with bipolar voltage < 0.5 mV. Early AF recurrence was defined as AF recurrence within 3 months after ablation and was assessed from follow‐up records. The 3‐month time point was selected because this follow‐up duration was uniformly available for all enrolled patients. Because this period overlaps with the conventional blanking period after AF ablation, the analysis of early AF recurrence was considered exploratory and was not intended to represent longer‐term post‐ablation recurrence.

### 2.5 Statistical Analysis

Continuous variables were summarized as mean ± standard deviation or median (interquartile range), as appropriate, and categorical variables as frequencies with percentages. Baseline characteristics were compared between the two groups using the Student's *t*‐test or Mann–Whitney *U* test for continuous variables and Fisher's exact test for categorical variables, as appropriate. The four AF likelihood categories output by AI‐ECG were dichotomized into the higher AF likelihood group (H or MH) and the lower AF likelihood group (ML or L). This dichotomization was used because the present study was exploratory and had a modest sample size, making category‐specific analyses potentially unstable, particularly in multivariable logistic regression models. In this study, the higher and lower AF likelihood groups refer to study‐defined groups based on AI‐ECG output from a sinus‐rhythm ECG and should not be interpreted as clinical risk strata, diagnostic correctness, or evidence for the absence of documented PAF.

Logistic regression analysis was performed to assess factors associated with assignment to the higher AF likelihood group among patients with documented PAF. Two prespecified multivariable models were constructed. The clinical model included sex, age, hypertension, and heart failure as prespecified clinically relevant covariates. The echocardiographic model included sex, age, left atrial volume index (LAVI), E/e', left ventricular mass index (LVMI), and tricuspid regurgitation (TR) grade, selected a priori to represent atrial remodeling, filling pressure/diastolic burden, ventricular remodeling, and valvular/hemodynamic burden [8].

Because all covariates included in the clinical model were available for all patients, the clinical model was fitted in the original dataset. For the echocardiographic model, missing covariate data were handled using multiple imputation with 20 imputed datasets. The imputation model included binary AF likelihood group assignment, variables in the echocardiographic model, and auxiliary variables including left atrial diameter index, left ventricular ejection fraction, heart failure, and NT‐proBNP. Estimates from the echocardiographic model were combined using Rubin's rules. A complete‐case analysis of the echocardiographic model was performed as a sensitivity analysis. The AUC was calculated for the clinical model in the original dataset and for the echocardiographic model in each imputed dataset, with AUCs averaged across imputations for the echocardiographic model. Confidence intervals for AUCs were estimated by bootstrap resampling. All statistical analyses were performed using SAS version 9.4 (SAS Institute Inc., Cary, NC, USA). Two‐sided *p* values < 0.05 were considered statistically significant.

### 2.6 Ethical Considerations

This study was conducted in accordance with the Declaration of Helsinki and the Ethical Guidelines for Medical and Biological Research Involving Human Subjects in Japan. The study protocol was approved by the Medical Ethics Committee of Oita University (approval No. 3145). Written informed consent was obtained from all participants before enrollment. This was a non‐interventional observational study, and the laboratory, electrocardiographic, and echocardiographic data used in the analysis were obtained as part of routine clinical care.

## 3 Results

**FIGURE 1:**
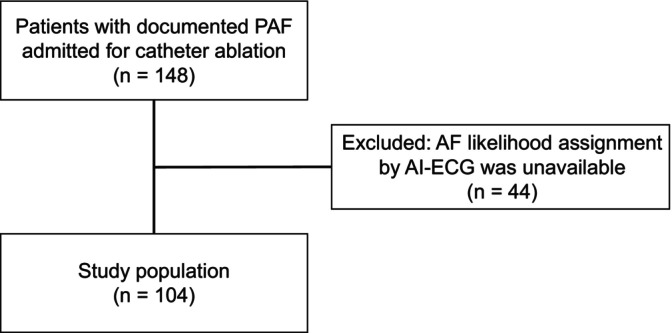
Flow diagram. Reasons for unavailable AF likelihood assignment by AI‐ECG included AF on the admission ECG (*n* = 22), PACs/PVCs (*n* = 11), pacemaker rhythm (*n* = 4), tachycardia (*n* = 2), AFL (*n* = 1), and other causes (*n* = 4). AF, atrial fibrillation; AFL, atrial flutter; AI‐ECG, artificial intelligence–enabled electrocardiography; PAC, premature atrial contraction; PAF, paroxysmal atrial fibrillation; PVC, premature ventricular contraction.

Among 148 patients with documented PAF, 44 were excluded because AF likelihood assignment by AI‐ECG was unavailable, most commonly due to AF or ectopic rhythm on the admission ECG (Figure 1). Baseline characteristics according to inclusion status in the AI‐ECG analysis are shown in Table S1. Age, sex, body mass index, most comorbidities, and medication use were not significantly different between included and excluded patients. Compared with included patients, excluded patients had higher NT‐proBNP levels (654.0 [306.5, 1722.5] vs. 136.0 [55.5, 324.0] pg/mL, *p* < 0.001), lower left ventricular ejection fraction (55.2% ± 9.8% vs. 62.3% ± 7.4%, *p* < 0.001), larger left atrial size, and a higher prevalence of mild or greater aortic regurgitation (32.5% vs. 16.5%, *p* = 0.042) and tricuspid regurgitation (85.0% vs. 63.1%, *p* = 0.015).

**FIGURE 2:**
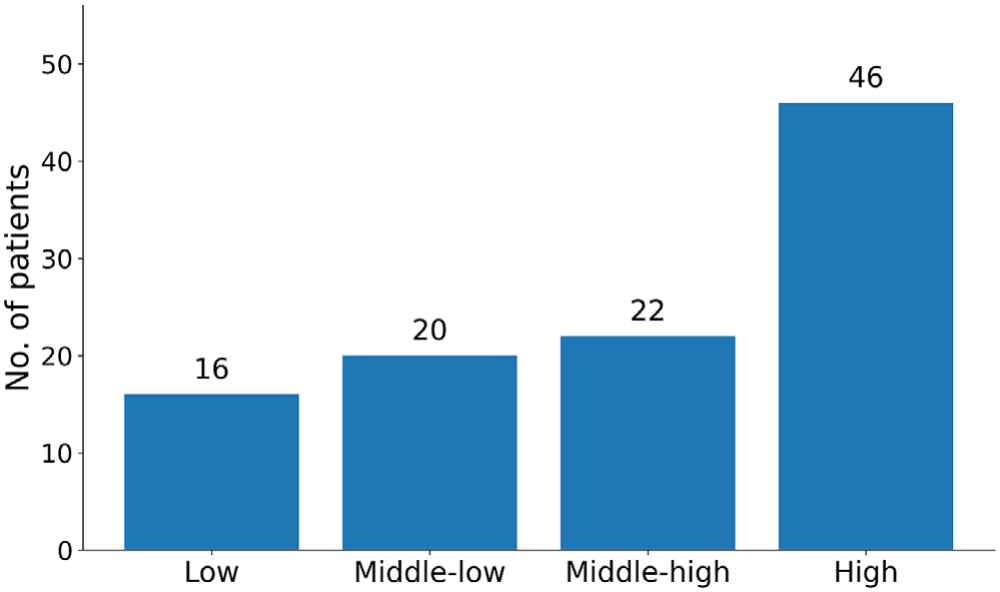
Distribution of AF likelihood categories assigned by AI‐ECG in patients with documented PAF. Bar graph showing the number of patients assigned by AI‐ECG to the low, middle‐low, middle‐high, and high AF likelihood categories during sinus rhythm. For subsequent analyses, high and middle‐high were grouped as the higher AF likelihood group, whereas middle‐low and low were grouped as the lower AF likelihood group. AI‐ECG, artificial intelligence‐enabled electrocardiography; PAF, paroxysmal atrial fibrillation.

Among the 104 patients with documented PAF, AI‐ECG assigned 36 patients to the lower AF likelihood group and 68 to the higher AF likelihood group (Figure 2). Baseline characteristics are summarized in Table 1, and the number of missing values for each variable is shown in Table S2. Most baseline variables were complete in both groups, and missing values were mainly limited to echocardiographic variables. Compared with the lower AF likelihood group, the higher AF likelihood group demonstrated a lower prevalence of hypertension (51.5% vs. 80.6%, *p* = 0.005) and lower use of dihydropyridine calcium channel blockers (29.4% vs. 52.8%, *p* = 0.032). Additionally, the higher AF likelihood group had lower LDL‐cholesterol levels (106.5 ± 27.6 vs. 117.4 ± 24.9 mg/dL, *p* = 0.045) and lower TSH levels (1.7 [1.1, 2.4] vs. 2.3 [1.4, 3.4] μIU/mL, *p* = 0.030). Regarding electrocardiographic and echocardiographic parameters, the higher AF likelihood group had a longer RR interval (1014.3 ± 161.4 vs. 929.7 ± 182.8 ms, *p* = 0.023), a lower LVMI (81.0 ± 25.0 vs. 95.8 ± 33.8 g/m^2^, *p* = 0.025), and a higher prevalence of mild or greater TR (70.1% vs. 47.2%, *p* = 0.033).

**TABLE 1:** Baseline characteristics according to AF likelihood group assigned by AI‐ECG.

	Lower AF likelihood group (*n* = 36)	Higher AF likelihood group (*n* = 68)	*p* value
Sex, female	14 (38.9)	26 (38.2)	1.000
Age, years	69.5 ± 8.8	69.0 ± 10.1	0.788
Body mass index, kg/m^2^	23.6 ± 3.3	23.5 ± 3.3	0.836
Comorbidities			
Hypertension	29 (80.6)	35 (51.5)	0.005
Diabetes	4 (11.1)	14 (20.6)	0.283
Dyslipidemia	19 (52.8)	41 (60.3)	0.533
Chronic kidney disease	10 (27.8)	10 (14.7)	0.123
Sleep apnea syndrome	2 (5.6)	7 (10.3)	0.492
Thyroid disorders	4 (11.1)	4 (5.9)	0.443
Coronary artery disease	2 (5.6)	7 (10.3)	0.492
Stroke	2 (5.6)	5 (7.4)	1.000
Heart failure	6 (16.7)	13 (19.1)	1.000
Current medications			
Antiarrhythmic drugs (Class I and IV)	7 (19.4)	21 (30.9)	0.348
Beta blocker	20 (55.6)	40 (58.8)	0.836
Amiodarone	5 (13.9)	6 (9.0)	0.510
ACE inhibitor/ARB/ARNI	20 (55.6)	29 (42.6)	0.223
MRA	5 (13.9)	10 (14.7)	1.000
Statin	13 (36.1)	39 (57.4)	0.063
Dihydropyridine CCBs	19 (52.8)	20 (29.4)	0.032
Laboratory test			
LDL‐cholesterol, mg/dL	117.4 ± 24.9	106.5 ± 27.6	0.045
HDL‐cholesterol, mg/dL	64.7 ± 15.6	63.3 ± 17.8	0.687
HbA1c, %	5.9 ± 0.6	5.9 ± 0.7	0.637
TSH, μIU/mL	2.3 [1.4, 3.4]	1.7 [1.1, 2.4]	0.030
FT4, ng/dL	1.3 [1.2, 1.4]	1.3 [1.2, 1.4]	0.548
eGFR, mL/min/1.73 m^2^	61.1 ± 31.6	62.3 ± 20.6	0.839
NT‐proBNP, pg/mL	98.0 [33.5, 386.5]	151.5 [88.0, 310.5]	0.132
12‐lead ECG			
RR interval, ms	929.7 ± 182.8	1014.3 ± 161.4	0.023
PR interval, ms	182.9 ± 33.6	174.3 ± 22.8	0.175
QRS interval, ms	118.3 ± 71.0	105.4 ± 35.4	0.311
QTc by Fridericia, ms	423.0 ± 31.7	420.3 ± 57.3	0.756
RV5 + SV1, mV	2.3 ± 0.8	2.5 ± 0.8	0.295
Transthoracic echocardiography			
Left ventricular ejection fraction, %	62.0 ± 5.9	62.2 ± 8.0	0.877
Left atrial diameter index, mm/m^2^	21.2 ± 3.7	21.4 ± 3.1	0.829
Left atrial volume index, mL/m^2^	35.9 ± 13.3	34.2 ± 11.3	0.574
Left ventricular mass index, g/m^2^	95.8 ± 33.8	81.0 ± 25.0	0.025
E/e' ratio	9.1 ± 3.9	8.6 ± 3.6	0.533
MR ≥mild	24 (66.7)	43 (64.2)	0.832
AR ≥mild	7 (19.4)	10 (14.9)	0.585
TR ≥mild	17 (47.2)	47 (70.1)	0.033
PR ≥mild	17 (47.2)	28 (41.8)	0.678

*Note:* Continuous variables are presented as mean ± standard deviation or median [25th percentile, 75th percentile], and categorical variables are presented as number (percentage).

Abbreviations: ACE, angiotensin‐converting enzyme; AI‐ECG, artificial intelligence‐enabled electrocardiography; AR, aortic regurgitation; ARB, angiotensin receptor blocker; ARNI, angiotensin receptor‐neprilysin inhibitor; CCB, calcium channel blocker; eGFR, estimated glomerular filtration rate; FT4, free thyroxine; HbA1c, hemoglobin A1c; MR, mitral regurgitation; MRA, mineralocorticoid receptor antagonist; NT‐proBNP, N‐terminal pro‐B‐type natriuretic peptide; PR, pulmonary regurgitation; TR, tricuspid regurgitation; TSH, thyroid‐stimulating hormone.

Additional procedural and follow‐up findings are shown in Table S3. The presence of left atrial low‐voltage area during the ablation procedure was not significantly different between the lower and higher AI‐ECG likelihood groups (6 [16.7%] vs. 8 [11.8%], *p* = 0.551). Early AF recurrence within 3 months after ablation was also not significantly different between the groups (3 [8.3%] vs. 3 [4.4%], *p* = 0.415).

In the clinical model, hypertension was inversely associated with assignment to the higher AF likelihood group (odds ratio [OR], 0.25; 95% confidence interval [CI], 0.10–0.66) (Table 2). The AUC of the clinical model was 0.651 (95% CI 0.603–0.805). In the echocardiographic model, higher LVMI was also inversely associated with assignment to the higher AF likelihood group (OR, 0.72 per 10 g/m^2^ increase; 95% CI, 0.57–0.90), whereas mild or greater TR showed a positive association (OR, 2.99; 95% CI 1.14–7.87) (Table 3). The AUC of the echocardiographic model was 0.718 (95% CI 0.650–0.843). In the complete‐case analysis, the inverse association for LVMI remained significant (OR, 0.68; 95% CI, 0.53–0.87), whereas the association for TR did not (OR, 2.04; 95% CI 0.67–6.24) (Table S4). The AUC for the complete‐case analysis of the echocardiographic model was 0.729 (95% CI 0.663–0.876).

**TABLE 2:** Clinical model for factors associated with assignment to the higher AF likelihood group.

	OR (95% CI)	*p* value
Sex, female	0.87 (0.34–2.25)	0.779
Age, per 1 year	1.00 (0.95–1.05)	0.930
Hypertension	0.25 (0.10–0.66)	0.005
Heart failure	0.94 (0.30–2.92)	0.920

*Note:* No missing data were present in the variables included in this model.

Abbreviations: AI‐ECG, artificial intelligence‐enabled electrocardiography; CI, confidence interval; OR, odds ratio.

**TABLE 3:** Echocardiographic model for factors associated with assignment to the higher AF likelihood group with multiple imputation.

	OR (95% CI)	*p* value
Sex, female	0.61 (0.21–1.76)	0.362
Age, per 1 year	0.99 (0.94–1.04)	0.667
LAVI, per 10 mL/m^2^	1.59 (0.87–2.88)	0.130
LV mass index, per 10 g/m^2^	0.72 (0.57–0.90)	0.005
E/e' ratio	0.94 (0.82–1.08)	0.394
TR ≥mild	2.99 (1.14–7.87)	0.027

*Note:* Missing covariate data were handled using multiple imputation with 20 datasets.

Abbreviations: AI‐ECG, artificial intelligence‐enabled electrocardiography; CI, confidence interval; LAVI, left atrial volume index; LV, left ventricular; OR, odds ratio; TR, tricuspid regurgitation.

## 4 Discussion

In this prospective observational study, approximately one‐third of patients with documented PAF were assigned by AI‐ECG to the lower AF likelihood group during sinus rhythm. The main findings were that hypertension and higher LVMI were inversely associated with assignment to the higher AF likelihood group, whereas mild or greater TR showed a possible positive association. Most previous studies of AI‐ECG during sinus rhythm have focused on identifying individuals with underlying AF or predicting incident AF [3, 4, 5, 6]. In contrast, the present study examined clinical and echocardiographic features associated with AI‐ECG‐based AF likelihood assignment in patients with documented PAF. The assignment of approximately one‐third of these patients to the lower AF likelihood group highlights a potential limitation of using a single sinus‐rhythm AI‐ECG assessment for AF screening. The observed associations between clinical and echocardiographic features and AF likelihood assignment by AI‐ECG further suggest that these features may be relevant when interpreting AI‐ECG output.

One of the most notable findings was that hypertension and higher LVMI were inversely associated with assignment to the higher AF likelihood group. Hypertension is a well‐established risk factor for AF, and hypertension‐related cardiac remodeling, including increased left ventricular mass, has been implicated in AF development and progression [9, 10]. In the present study, however, hypertension and higher LVMI were inversely associated with assignment to the higher AF likelihood group. These results suggest that the specific AI‐ECG model employed in this study may be less likely to assign patients with ECG changes related to hypertensive remodeling to the higher AF likelihood group. This interpretation is biologically plausible because prior AI‐ECG studies have suggested that AF prediction using sinus‐rhythm ECGs relies importantly on the P‐wave and adjacent PR region [11, 12], whereas hypertension has been associated with atrial remodeling and abnormal P‐wave indices [13, 14]. Accordingly, hypertension‐related waveform changes may obscure the ECG patterns that lead this system to assign patients to the higher AF likelihood group. However, because we did not analyze specific waveform features and the underlying algorithm is proprietary, this interpretation remains speculative.

Another notable finding was the association between mild or greater TR and assignment to the higher AF likelihood group in the primary analysis after multiple imputation, although this association was not statistically significant in the complete‐case analysis. This may be informative because atrial or functional TR is increasingly recognized in patients with AF and has been linked to right atrial enlargement and tricuspid annular remodeling [15, 16, 17]. LAVI was not significantly associated with assignment to the higher AF likelihood group in the present analysis. However, this finding does not exclude a potential role of left atrial remodeling in AI‐ECG‐based AF likelihood assignment. Prior studies have shown that larger left atrial size or volume is associated with AF progression and with more sustained AF [18, 19, 20].

The longer RR interval observed in the higher AF likelihood group may also warrant attention. A slower heart rate may increase the visibility of subtle atrial electrical features [12, 21], although this interpretation remains speculative. Differences in LDL‐cholesterol and TSH should also be interpreted cautiously. Although lower LDL‐cholesterol has been linked to higher AF risk in prior epidemiologic studies [22, 23, 24], the clinical significance of the LDL‐cholesterol and TSH findings in the present cohort remains unclear.

These findings suggest that AF likelihood assignment by AI‐ECG may not simply correspond to structural remodeling related to AF. Although hypertension, increased LVMI, and TR are all linked to AF development or progression, their associations with AI‐ECG output were not uniform in the present study.

Several limitations should be acknowledged. First, this was a single‐center study with a modest sample size and included only patients with documented PAF referred for catheter ablation. The findings therefore may not be generalizable to populations with unknown AF status, in whom AI‐ECG would be used for AF screening or risk assessment. Second, 44 patients were excluded because AI‐ECG likelihood assignment was unavailable, primarily due to AF (*n* = 22), other arrhythmias, or pacemaker rhythms on the admission ECG. Compared with the patients included in the analysis, these excluded patients had higher NT‐proBNP levels and less favorable echocardiographic findings, suggesting a less favorable cardiac profile. Therefore, the present findings regarding AI‐ECG output may not be generalizable to the entire PAF population, particularly to patients with more advanced structural or functional abnormalities.

Third, the present findings may be specific to the AI‐ECG algorithm used in the FCP‐9900Ai system. Differences in clinical characteristics between patients with and without AF in the model‐development dataset could have influenced the features learned by the algorithm and the resulting AF likelihood assignment. External validation using other AI‐ECG systems and populations is needed. Fourth, some echocardiographic variables, particularly LAVI, had missing values. Although multiple imputation and complete‐case analyses were performed, residual uncertainty related to missing data remains. Fifth, echocardiographic data were obtained from outpatient examinations performed within 6 months before admission, whereas AF likelihood assignment by AI‐ECG was based on the admission ECG. This temporal gap may have affected the associations between echocardiographic findings and AF likelihood assignment by AI‐ECG. Sixth, the AI‐ECG likelihood categories were assigned from a single 12‐lead ECG obtained at admission. A previous study using the FCP‐9900Ai system reported within‐patient variability in the four‐level AI output, including variation related to ECG recording conditions [25]. Because repeated ECG assessment was not performed, we could not evaluate the stability of AI‐ECG likelihood categories within individual patients. Seventh, AF burden and duration of PAF were not systematically available in this study. Standardized prolonged rhythm monitoring was not performed in all patients, and the timing of the first documented PAF episode could not be reliably determined in some patients. Therefore, we could not determine whether AI‐ECG likelihood assignment was associated with AF burden or disease duration among patients with documented PAF. Finally, we did not perform detailed ECG waveform analyses, such as assessment of P‐wave morphology, duration, or PR‐related features. Therefore, the specific ECG waveform features associated with AI‐ECG output could not be determined.

## 5 Conclusions

A lower AF likelihood assignment by AI‐ECG was not uncommon among patients with documented PAF during sinus rhythm. Hypertension and higher LVMI were inversely associated with assignment to the higher AF likelihood group, whereas mild or greater TR showed a possible positive association. These findings suggest heterogeneity in AF likelihood assignment by AI‐ECG among patients with documented PAF. Further studies are needed to clarify which patient characteristics are associated with AI‐ECG output and how this output should be interpreted when AI‐ECG is used for AF assessment.

## Author Contributions

Hiroki Sato and Naohiko Takahashi conceived and designed the study. Hiroki Sato conducted the study, analyzed and interpreted the data, and drafted the manuscript. Akiko Baba, Yuki Kubota, Kei Hirota, Hidekazu Kondo, Akira Fukui, Tetsuji Shinohara, and Yasushi Teshima contributed to patient enrollment and data collection and critically revised the manuscript for important intellectual content. Nozomi Kodama, Miho Miyoshi, and Tomoko Fukuda contributed to the acquisition, assessment, and interpretation of electrocardiographic and echocardiographic data and critically revised the manuscript for important intellectual content. Naohiko Takahashi supervised the study and critically revised the manuscript for important intellectual content. All authors approved the final version of the manuscript and agreed to be accountable for all aspects of the work.

## Funding

This study was supported by Fukuda Denshi Co. Ltd. The study was conducted under institutional conflict‐of‐interest management procedures at Oita University. In accordance with the study protocol, the research was conducted fairly and appropriately without undue influence from the funding source.

## Ethics Statement

This study was approved by the Medical Ethics Committee of Oita University (Approval No. 3145), and all participants provided written informed consent.

## Consent

All participants provided written informed consent.

## Conflicts of Interest

This study received funding from Fukuda Denshi Co. Ltd. The authors declare no other conflicts of interest.

## Supporting information


**Table S1:** Baseline characteristics according to inclusion status in the AI‐ECG analysis.
**Table S2:** Number of missing values for baseline characteristics among included patients stratified by AI‐ECG–assigned AF likelihood group and excluded patients.
**Table S3:** Additional procedural and follow‐up findings according to AI‐ECG likelihood group.
**Table S4:** Complete‐case analysis of the echocardiographic model for factors associated with assignment to the higher AF likelihood group.

## Data Availability

The data that support the findings of this study are available from the corresponding author upon reasonable request. The data are not publicly available due to privacy or ethical restrictions.
