# Supplementary material for: Clinical and Echocardiographic Factors Associated With AI‐Estimated Atrial Fibrillation Likelihood During Sinus Rhythm in Patients With Documented Paroxysmal Atrial Fibrillation
Source: J Arrhythm. 2026 Aug 3;42(4):e70439. doi: 10.1002/joa3.70439 (PMC13430280; doi:10.1002/joa3.70439)
Supplement: Supplementary file 1 — Table S1: Baseline characteristics according to inclusion status in the AI‐ECG analysis. Table S2: Number of missing values for baseline characteristics among included patients stratified by AI‐ECG–assigned AF likelihood group and excluded patients. Table S3: Additional procedural and follow‐up findings according to AI‐ECG likelihood group. Table S4: Complete‐case analysis of the echocardiographic model for factors associated with assignment to the higher AF likelihood group. [file JOA3-42-e70439-s001.pdf]

Supplementary Table 1. Baseline characteristics according to inclusion status in the AI-ECG analysis

	Included (n = 104)	Excluded (n = 44)	p value
Sex, female	40 (38.5)	18 (40.9)	0.854
Age, years	69.1 ± 9.6	69.4 ± 12.1	0.885
Body mass index, kg/m^2	23.5 ± 3.3	22.7 ± 3.4	0.178
Comorbidities			
Hypertension	64 (61.5)	24 (54.5)	0.467
Diabetes	18 (17.3)	5 (11.4)	0.461
Dyslipidemia	60 (57.7)	20 (45.5)	0.208
Chronic kidney disease	20 (19.2)	9 (20.5)	1.000
Sleep apnea syndrome	9 (8.7)	2 (4.5)	0.507
Thyroid disorders	8 (7.7)	5 (11.4)	0.529
Coronary artery disease	9 (8.7)	0 (0.0)	0.058
Stroke	7 (6.7)	4 (9.1)	0.733
Heart failure	18 (17.3)	6 (13.6)	0.636
Current medications			
Antiarrhythmic drugs (Class I and IV)	28 (26.9)	6 (13.6)	0.090
Beta blocker	60 (57.7)	28 (63.6)	0.584
Amiodarone	11 (10.7)	7 (15.9)	0.414
ACE inhibitor / ARB / ARNI	49 (47.1)	14 (31.8)	0.103
MRA	15 (14.4)	7 (15.9)	0.805
Statin	52 (50.0)	15 (34.1)	0.104
Dihydropyridine CCBs	39 (37.5)	13 (29.5)	0.452
Laboratory test			
LDL-cholesterol, mg/dL	110.3 ± 26.9	108.1 ± 23.9	0.625
HDL-cholesterol, mg/dL	63.8 ± 17.0	63.5 ± 14.8	0.917
HbA1c, %	5.9 ± 0.7	5.9 ± 0.5	0.977
TSH, µIU/mL	1.8 [1.2, 2.8]	1.9 [1.2, 3.5]	0.485
FT4, ng/dL	1.3 [1.2, 1.4]	1.3 [1.2, 1.5]	0.759
eGFR, mL/min/1.73 m^2	61.9 ± 24.8	59.1 ± 24.5	0.527
NT-proBNP, pg/mL	136.0 [55.5, 324.0]	654.0 [306.5, 1722.5]	<0.001
Transthoracic echocardiography			
Left ventricular ejection fraction, %	62.3 ± 7.4	55.2 ± 9.8	<0.001
Left atrial diameter index, mm/m^2	21.3 ± 3.3	23.1 ± 3.5	0.006
Left atrial volume index, mL/m^2	34.8 ± 12.0	40.8 ± 13.2	0.025
Left ventricular mass index, g/m^2	86.1 ± 29.0	91.6 ± 28.2	0.304
E/e′ ratio	8.8 ± 3.7	9.3 ± 3.9	0.462
MR ≥ mild	67 (65.0)	32 (80.0)	0.107
AR ≥ mild	17 (16.5)	13 (32.5)	0.042

TR ≥ mild	65 (63.1)	34 (85.0)	0.015
PR ≥ mild	45 (43.7)	21 (52.5)	0.357

ACE, angiotensin-converting enzyme; AI-ECG, artificial intelligence-enabled electrocardiography; ARB, angiotensin receptor blocker; ARNI, angiotensin receptor-neprilysin inhibitor; MRA, mineralocorticoid receptor antagonist; CCB, calcium channel blocker; HbA1c, hemoglobin A1c; TSH, thyroid-stimulating hormone; FT4, free thyroxine; eGFR, estimated glomerular filtration rate; NT-proBNP, N-terminal pro-B-type natriuretic peptide; MR, mitral regurgitation; AR, aortic regurgitation; TR, tricuspid regurgitation; PR, pulmonary regurgitation.

Continuous variables are presented as mean ± standard deviation or median [25th percentile, 75th percentile], and categorical variables are presented as number (percentage).

The 12-lead ECG parameters were not summarized because rhythm and pacing status varied in the excluded group.

Supplementary Table 2. Number of missing values for baseline characteristics among included patients stratified by AI-ECG–assigned AF likelihood group and excluded patients

	Lower AF likelihood group (n = 36)	Higher AF likelihood group (n = 68)	Excluded (n = 44)
Sex	0	0	0
Age	0	0	0
BMI	1	0	0
Comorbidities			
Hypertension	0	0	0
Diabetes	0	0	0
Dyslipidemia	0	0	0
Chronic kidney disease	0	0	0
Sleep apnea syndrome	0	0	0
Thyroid disorders	0	0	0
Coronary artery disease	0	0	0
Stroke	0	0	0
Heart failure	0	0	0
Current medications			
Antiarrhythmic drugs (Class I and IV)	0	0	0
Beta blocker	0	0	0
Amiodarone	0	0	0
ACE inhibitor / ARB / ARNI	0	0	0
MRA	0	0	0
Statin	0	0	0
Dihydropyridine CCBs	0	0	0
Laboratory test			
LDL-cholesterol	0	1	0
HDL-cholesterol	0	1	0
HbA1c	0	0	0
TSH	0	0	0
FT4	0	0	0
eGFR	0	0	0
NT-proBNP	0	0	0
12-lead ECG			
RR interval	0	0	–
PR interval	0	0	–
QRS interval	0	0	–
QTc by Fridericia	0	0	–
RV5 + SV1	0	0	–

Transthoracic echocardiography			
Left ventricular ejection fraction	0	1	4
Left atrial diameter index	2	2	4
Left atrial volume index	9	13	10
Left ventricular mass index	0	2	4
E/e′ ratio	0	2	4
MR ≥ mild	0	1	4
AR ≥ mild	0	1	4
TR ≥ mild	0	1	4
PR ≥ mild	0	1	4

ACE, angiotensin-converting enzyme; AI-ECG, artificial intelligence-enabled electrocardiography; ARB, angiotensin receptor blocker; ARNI, angiotensin receptor-neprilysin inhibitor; BMI, body mass index; MRA, mineralocorticoid receptor antagonist; CCB, calcium channel blocker; HbA1c, hemoglobin A1c; TSH, thyroid-stimulating hormone; FT4, free thyroxine; eGFR, estimated glomerular filtration rate; NT-proBNP, N-terminal pro-B-type natriuretic peptide; MR, mitral regurgitation; AR, aortic regurgitation; TR, tricuspid regurgitation; PR, pulmonary regurgitation.

Supplementary Table 3. Additional procedural and follow-up findings according to AI-ECG likelihood group

	Lower AF likelihood group (n = 36)	Higher AF likelihood group (n = 68)	p value
Presence of left atrial low-voltage area	6 (16.7)	8 (11.8)	0.551
Early AF recurrence within 3 months after ablation	3 (8.3)	3 (4.4)	0.415

AF, atrial fibrillation; AI-ECG, artificial intelligence-enabled electrocardiography.

Values are presented as number (percentage). Left atrial low-voltage area was assessed during the ablation procedure and defined as an area with bipolar voltage <0.5 mV.

Supplementary Table 4. Complete-case analysis of the echocardiographic model for factors associated with assignment to the higher AF likelihood group

	OR (95% CI)	p value
Sex, female	0.56 (0.17–1.89)	0.350
Age, per 1 year	1.00 (0.94–1.07)	0.937
LAVI, per 10 mL/m^2	1.63 (0.88–3.63)	0.119
LV mass index, per 10 g/m^2	0.68 (0.53–0.87)	0.003
E/e′ ratio	0.93 (0.79–1.09)	0.367
TR ≥ mild	2.04 (0.67–6.24)	0.212

Analyses were performed using complete-case data.

AI-ECG, artificial intelligence-enabled electrocardiography; CI, confidence interval; LAVI, left atrial volume index; LV, left ventricular; OR, odds ratio; TR, tricuspid regurgitation.
